# Tale of Two Centres—Exploring Bedside Challenges to Implementing a Clinical Quality Registry for Abdominal Wall Hernias in Metropolitan Australia

**DOI:** 10.1111/ans.70694

**Published:** 2026-04-24

**Authors:** Edward Young, Doug Fenton‐Lee, Julian Süsstrunk, Jorgen Ferguson, Matthew Amprayil, Yijie Yin, Rabieh Abu Assi, Alex Karatassas, Sanjeev Khurana, Chrys Hensman, Andrew Tse, Hamish Urquhart, Reginald V. N. Lord, Jean Wong, Darren Tonkin, Markus Trochsler, Guy J. Maddern

**Affiliations:** ^1^ Department of Surgery, The Queen Elizabeth Hospital Adelaide University (The University of Adelaide) Adelaide South Australia Australia; ^2^ The Basil Hetzel Institute Woodville South South Australia Australia; ^3^ Department of Surgery St Vincent's Public and Private Hospital Sydney Darlinghurst New South Wales Australia; ^4^ School of Medicine, Notre Dame University Sydney New South Wales Australia; ^5^ Department of Visceral Surgery Clarunis University Digestive Health Care Center, St. Clara Hospital and University Hospital Basel Basel Switzerland; ^6^ Department of Surgery Women's and Children's Hospital North Adelaide South Australia Australia; ^7^ Data Dissect Daw Park South Australia Australia; ^8^ Monash University, Swinburne University of Technology, University of Oceania Melbourne Victoria Australia

**Keywords:** clinical quality registry, cohort, database, hernia, literature review

## Abstract

**Background:**

In an effort to improve hernia care and mesh product surveillance, the Australia and New Zealand Hernia Society (ANZ Hernia) has been setting up a prototype binational learning healthcare system virtual clinical quality registry (CQR) for clinical use. The aim of this study was to assess the feasibility for clinicians to enrol patients for a prototype hernia CQR in a typical clinical environment across two metropolitan centres in Australia.

**Methods:**

All English‐speaking adult patients with capacity to consent and who were being managed for abdominal wall hernias by participating clinicians at the Queen Elizabeth Hospital (Adelaide) or at St Vincent's Private Hospital (Sydney) during specified periods were eligible for inclusion. Participating clinicians were recruited and encouraged to provide feedback. The total number of eligible patients for each recruitment period was determined from clinical records. The literature was reviewed to summarise existing hernia registries.

**Results:**

Over a continuous 6‐month recruitment period, the conditional recruitment rate of eligible patients encountered by participating clinicians was 34.7% (25 of 74) at TQEH (07/08/2024–02/02/2025) and 48.7% (58 of 119) at SVPHS (15/08/2025–10/02/2025). In the absence of incentives and sustained funding, clinician goodwill alone was insufficient to achieve the minimal standards required by the Australian Commission on Safety and Quality in Health Care for CQRs. An in‐depth analysis of the CQR was conducted.

**Conclusion:**

The prototype ANZ Hernia CQR would benefit from a redesign to address the structural issues identified, if meaningful system‐wide quality improvement is to be delivered across Australia and New Zealand as envisioned.

**Abbreviations**
ABS SEIFA: Australian Bureau of Statistics Socio‐Economic Indexes for Areas
ACHQC: Abdominal Core Health Quality Collaborative
AI: artificial intelligence
ANZ Hernia: Australia and New Zealand Hernia Society
ANZASM: Australia and New Zealand Audit of Surgical Mortality
AOA: Australian Orthopaedic Association
AOANJRR: Australian Orthopaedic Association National Joint Replacement Registry
ASA: American Society Anesthesiologists
CALD: culturally and linguistically diverse
CALHN: Central Adelaide Local Health Network
CPD: continued professional development
CQR: clinical quality registry
ELAR: endoscopic linea alba reconstruction
eMILOS: endoscopic mini or less open sublay
eTEP: extended totally extraperitoneal
HREC: Human Research Ethics Committee
ICMJE: International Committee of Medical Journal Editors
IPOM: intraperitoneal onlay mesh
LHS: learning healthcare system
LIRA: laparoscopic intracorporeal rectus aponeuroplasty
MBS: Medical Benefits Schedule
MEDSAFE: Medicines and Medical Devices Safety Authority
MILOS: mini or less open sublay
MRFF: medical research future fund
NHI: National Health Index
PHARMAC: Pharmaceutical Management Agency
PICF: patient information consent form
RACS: Royal Australasian College of Surgeons
RCT: randomised controlled trials
reTEP: robotic extended totally extraperitoneal
rTAPP: robotic transabdominal preperitoneal
rTEP: robotic totally extraperitoneal
SCOLA: subcutaneous onlay laparoscopic approach
SVPHS: St Vincent's Private Hospital Sydney
TAPP: transabdominal preperitoneal
TARM: transabdominal retromuscular repair
TEP: totally extraperitoneal
TESAR: totally endoscopic sublay anterior repair
TGA: Therapeutic Goods Administration
TIPP: transinguinal preperitoneal
TQEH: the Queen Elizabeth Hospital
TREPP: transrectus peritoneal

## 1 Introduction

Each year, 100 000 abdominal wall hernia operations are performed in Australia [1], with over 80 000 hernia meshes implanted [2]. Hernia repairs and mesh devices have traditionally been evaluated via 30‐day outcomes or 1‐year endpoints, yet increasing evidence suggests that this is inadequate in capturing complications that develop years after index surgery [2, 3]. Some authors have advocated that hernia care follow‐up needs to continue for 5–10 years at a minimum, and perhaps even up to 50 years if true complication rates are to be determined [3]. To adequately capture long‐term outcomes of hernias, hernia registries have been established around the world, with the earliest being the Swedish Hernia Registry [4].

High‐quality hernia registries can capture longitudinal data on hernia care and monitor mesh devices, as demonstrated by the prominent role of the Danish and German Hernia Registries in the worldwide recall of Physiomesh [5, 6]. Registries of sufficient size, completeness and duration may be suitable for registry‐based randomised controlled trials (RCT), overcoming the cost and duration limitations present in traditional RCTs [7]. In an effort to improve hernia care and surveillance, the Australia and New Zealand Hernia Society (ANZ Hernia) has been setting up a prototype binational clinical quality registry (CQR) for clinical use [8].

The foundation of any successful CQR is data quality [9]. CQRs with missing data or non‐representative populations may provide misleading reassurances and lead to incorrect conclusions [10]. For CQR data to be considered high quality, at least 95% of the target population needs to be recruited [11]. Of the 16 surgical CQRs currently registered with the Australian Commission on Safety and Quality in Health Care (the Commission), only two have achieved and maintained > 95% patient inclusion [10, 12]. These are the Australian Orthopaedic Association National Joint Replacement Registry (AOANJRR) (98.8% inclusion) [13], and the Royal Australasian College of Surgeons Australia and New Zealand Audit of Surgical Mortality (ANZASM) (~100% inclusion) [14]. Both are primarily funded by the Australian Government Department of Health, Disability and Aging, with oversight by the respective surgical societies and inclusion within annual continued professional development (CPD) requirements. High‐quality CQRs around the world have used similar incentive measures [10, 15, 16]. It is likely similar processes are required for a hernia CQR, though the current Australian landscape of clinician involvement and barriers to patient recruitment are unclear.

The aim of this study was to assess the feasibility for clinicians to enrol patients for a prototype hernia clinical quality registry in typical clinical environments across two metropolitan centres in Australia.

## 2 Methods

### 2.1 The Registry

The ANZ Hernia society is a binational collective of surgeons with an interest in the diagnosis and treatment of abdominal wall defects and hernias [17]. Established since 2021 and endorsed by the Royal Australasian College of Surgeons (RACS), the ANZ Hernia society fosters education, training and translational research in hernia care.

The ANZ Hernia Learning Healthcare System Virtual Clinical Quality Registry (ANZ Hernia CQR) is a prototype cloud‐based platform designed to record pertinent information on hernia care in Australia and New Zealand. With reference to the United States' Abdominal Core Health Quality Collaborative (ACHQC) framework and the requirements outlined by the Commission [16], a panel of consultant surgeons specialising in hernias was established by the ANZ Hernia Database Subcommittee, and registry questions relevant to the Australia and New Zealand clinical landscape were selected by the panel via a Delphi process. The Registry is currently funded and maintained in whole by the ANZ Hernia society. The Registry is hosted on Data Dissect cloud servers (Data Dissect Pty Ltd., Australia) with integrated enterprise‐strength cybersecurity measures.

To address the limitations of traditional clinical quality registries, the Registry has been designed with a Learning Healthcare System (LHS) at its core. Coined in 2007 by the United States National Academy of Sciences, LHS is an overarching term for the next generation of healthcare data systems for bedside care and clinical research with real‐time feedback, allowing healthcare to be theoretically delivered in a more efficient and cost‐effective manner [18, 19, 20, 21, 22]. The Registry may be considered to be ‘virtual’ or a by‐product of an LHS, as information is collected as part of normal patient care. The system automatically outputs research summaries based on the audit questions inputted, with a miniaturised example of this being live status boards of hospital bed flow [23]. The development of LHS has accelerated in recent years due to the exponential rise in sophistication of neural network artificial intelligence (AI) with deep learning capabilities, allowing vast quantities of digital data to be processed and summarised within seconds to minutes [24, 25, 26].

The Registry consists of five parts, namely patient registration, baseline, planning, treatment and follow‐up (Supplementary S1–S5), structured according to a clinical pathway typically undertaken by a multidisciplinary team (MDT) to manage complex abdominal wall hernias [27]. Registry questions are a mix of multiple choices or free text. The Registry can auto‐send patient‐reported outcome measurements (PROMs) and patient‐reported experience measures (PREMs) to facilitate follow up.

### 2.2 Ethics and Registration

This study was conducted at the Queen Elizabeth Hospital (TQEH) in South Australia and St Vincent's Private Hospital Sydney (SVPHS) in New South Wales. Each site had a designated principal investigator (PI) as the site lead.

A third site in New South Wales and a fourth site in Victoria had originally been included in the 2021 ethics application to the local Human Research Ethics Committee (HREC), though site participation was withdrawn following the untimely death of a PI and local administrative issues. Changes in research personnel and waning interest further interrupted study recruitment in 2022 and 2023. In an attempt to address these critical issues, the study protocol was updated to focus on staff engagement, ergonomics and the process of recruiting patients into the CQR. Further recruitment sites were considered but deemed not time‐efficient for the task on hand, as site‐specific HREC approvals were required but had not been obtained yet.

The updated study protocol was approved by the Central Adelaide Local Health Network (CALHN) HREC for TQEH (Reference No. 15562), with reciprocal ethics approved by St Vincent's Hospital HREC for extension to SVPHS. Due to ethical approval constraints, public patients at St Vincent's Hospital were not available for recruitment during the study period. Ethics approval was only granted for an ‘opt‐in’ system, due to the use and storage of identifiable patient information on non‐healthcare facility data servers. This required all patients to provide informed consent prior to enrolment and data entry.

This study was not preregistered due to its exploratory nature. This study was performed in accordance with the ethical standards laid down in the Declaration of Helsinki. This study was prepared and reported according to the CONSORT 2010 Statement with Extension to Randomised Pilot and Feasibility Trial Guidelines, with non‐applicable items discarded as per Lancaster and Thabane [28].

### 2.3 Setting and Location

TQEH is a 355‐bed, acute and sub‐acute care public teaching hospital that provides inpatient, outpatient, elective and emergency health services to a population of more than 250 000 people living primarily in Adelaide's western suburbs [29]. Abdominal wall hernias are managed primarily by general surgery, namely the Upper Gastrointestinal (Upper GI) and the Colorectal units. Emergency general surgery is provisioned by these two units on an alternating 24‐h on‐call roster system. Cost of treatment is fully covered by Medicare, Australia's universal healthcare scheme.

SVPHS is a 310‐bed acute care tertiary private hospital, adjacent to its 379‐bed public counterpart. SVPHS accepts patients across New South Wales and provides highly specialised surgical and medical services [30]. Abdominal wall hernias are managed electively and operated on by consultants specialising in general and colorectal surgery. Cost of treatment is predominantly covered by patients' private health insurance or equivalent, with any remaining gaps paid directly by patients. Any patients who could not afford private healthcare were treated publicly and were not included in this study.

### 2.4 Participating Clinicians

The study was announced and advertised to members of the general surgical teams by the respective PIs at each site. There were no financial incentives or remuneration for involvement. Participating clinicians were advised that their efforts would be acknowledged or listed as authors, depending on the level of contribution as defined by the International Committee of Medical Journal Editors (ICMJE) guidelines [31]. All participating clinicians were given training on how to consent patients, use the registry and input data into the registry via 1:1 training sessions by the PIs.

### 2.5 Inclusion and Exclusion Criteria

All English‐speaking adult patients (age ≥ 18 years) who had capacity to consent and were being seen or treated for abdominal wall hernias by participating clinicians at the approved healthcare facilities during the specified study period were eligible for inclusion within this study (Table 1). Patients were excluded if they did not meet these criteria. All inguinal, femoral, primary ventral, and incisional hernias were eligible for inclusion. Eligibility criteria were not altered at any time during the study.

**TABLE 1:** Inclusion and exclusion criteria.

Inclusion	Exclusion
Adult, age ≥ 18 years Abdominal wall hernia Clinical encounter on system (outpatient, elective, emergency) TQEH or SVPHS Capacity to consent Proficient with conversational English	Non‐abdominal wall hernia (e.g., hiatus, diaphragmic) Non‐clinical encounter (e.g., visitor) Patients from other hospitals (e.g., referrals for MDT meeting) No capacity to consent

Abbreviations: MDT, multidisciplinary team; SVPHS, St Vincent's Private Hospital Sydney; TQEH, the Queen Elizabeth Hospital.

Power calculations were not applicable, as the purpose of the study was to observe how many patients could be recruited, relative to total eligible patients encountered by participating clinicians. Target for recruitment was 95% of total eligible patients encountered.

### 2.6 Patient Consent

All patients were required to provide informed consent using an approved standardised Patient Information Consent Form (PICF). The PICF presented the key details of the study in lay language and was complemented with an infographic handout (Supplementary S6). The consent form was available in paper format or as a digital form on the registration page of the Registry. Due to the lack of professional translation services, all materials were only available in English. To ensure patients fully understood the materials, patients were restricted to English‐speaking only with the exclusion of culturally and linguistically diverse (CALD) patients. Consent was performed by participating clinicians at the time of the clinical encounter. Patients were given time and opportunity to consider their decision.

Participating medical staff were allowed to obtain informed consent from patients in any format or delivery that they deemed fit for the situation. Patients were allowed to be recruited retrospectively after their surgical operation or during post‐operative follow up. Participants were allowed to withdraw from the study at any time by communicating with the PIs via contact details listed in the PICF.

### 2.7 Study Period

The study period was from Jul 2024 to Aug 2025. An initial short 2‐week period in Jul 2024 was scheduled at TQEH to test run the system and to obtain general feedback from participating clinicians. Recruitment periods were divided into 6‐month blocks to minimise the influence of staff changes. The study was to be terminated individually at each site if no patient was recruited over a 2‐week period.

### 2.8 Data and Statistics

Problems identified during recruitment were thematically grouped and tabulated.

Data were exported in deidentified form from the Registry. Collected demographics included gender, age, and postcode. Postcode was cross‐referenced with the Australian Bureau of Statistics Socio‐Economic Indexes for Areas (ABS SEIFA) to estimate the level of socio‐economic disadvantage [32].

The total number of eligible patients was determined from clinical activity during the study periods, provided medical records were accessible. The number of eligible patients encountered by each participating medical staff was determined based on who made the initial entry and assessment in medical records. If two or more participating medical staff were involved in separate encounters, the patient was assigned to the most senior medical officer to avoid double counting.

All patients were categorised into clinic attendance with non‐operative management, clinical attendance with elective bookings made, and emergency admissions without any prior elective booking. Subcategories included whether surgery had taken place during the study period and whether any post‐operative follow‐up occurred.

Statistical analysis was performed using RStudio software (v2024.09.1.394, Posit Team, United States) [33], running R language (v4.4.2, R Core Team, Austria) [34]. Chi‐squared test was performed in base R to compare demographics between subgroups and study sites. Significance level was defined as p<0.05.

### 2.9 Literature Review

PubMed and Scopus databases were systematically searched to identify existing hernia registries, using keyword combinations of dataset, registry, hernia, implement, recruit or their derivatives (Supplementary S7). Key aspects of the respective hernia registries were summarised.

## 3 Results

Patient recruitment at the TQEH had three distinct periods (Table 2). The first period was the planned 2‐week testing phase, lasting from 22 July 2024 to 2 August 2024, and involved six members of the surgical team (17.6%). The second period lasted from 7 August 2024 to 2 February 2025, and involved 12 members (35.3%). The third period began on 2 February 2025, but was terminated early on 16 February 2025 due to low interest from clinicians and no patient recruitment. No patient withdrew from the study at any point.

**TABLE 2:** Breakdown of participating clinicians by role and recruitment period.

Site	Period	Consultants	Fellows	Registrars	RMOs	Interns	Total	%
TQEH	22/07/2024–02/08/2024	2/14	2/4	1/6	1/6	0/4	6/34	17.6
	07/08/2024–02/02/2025	2/14	2/4	4/6	2/6	2/4	12/34	35.3
	02/02/2025–16/02/2025	2/14	1/4	1/6	0/6	0/4	4/34	11.8
SVPHS	15/08/2024–10/02/2025	4/12	1/1	—	—	—	5/13	38.4

Abbreviations: RMO, resident medical officer; SVPHS, St Vincent's Private Hospital Sydney; TQEH, the Queen Elizabeth Hospital.

Recruitment at SVPHS began on 15 August 2024 after HREC approval. To ensure data was comparable to the second period at TQEH, a similar 6‐month period was defined and data was utilised up to 10 February 2025. SVPHS was not impacted by staff changes.

In the first recruitment period at TQEH, a total of four patients were documented to have been approached, with two declining to participate. Numerous issues were raised by the participating clinicians (Table 3). Three distinct themes were identified, namely the difficulty of staff to participate in recruitment due to competing activities (e.g., fellowship exams) or non‐hernia interests, difficulties with consent and registration (e.g., slow online interface, long registration form), and problems with integrating into clinical workflow (e.g., clinical work taking priority over research tasks). To address these issues and maximise patient engagement for the second recruitment period, participating clinicians were advised to focus on consenting patients for the registry via paper forms at time of encounter and to record pertinent information within clinical records. Online registration was completed retrospectively by the clinician or the PI, on behalf of patients.

**TABLE 3:** Problems identified at the Queen Elizabeth Hospital, from 22/07/2024 to 02/08/2024 (first period), grouped by theme.

Theme	Problem	Solution
Medical staff participation	Fellows cited being busy with clinical workload, on call or competing research projects. Registrars often were preparing or sitting clinical/fellowship exams. Some cited non‐interest in hernia field. Junior staff cited low interest in engaging with a project when they were going to be rotating soon/leaving the unit soon.	Targeted recruitment of medical staff over a complete period of hospital rotation Increased focus and efforts on the limited number of highly motivated medical officers who were willing to engage and participate in the project. Engage with consultants and fellows to have a top‐down approach to encourage involvement and recruitment of patients for projects Regular contact between principle investigators and participating staff members to review and troubleshoot any potential issues that have arisen
Consent and registration	Patients were more receptive of the project when informed consent was provided at time of encounter, as opposed to filling out a digital form at a later date. Photocopying signed consent forms was inconvenient during clinical encounters. A portion of patients were of elderly age and did not appear to be sufficiently proficient with digital technology to use QR codes. Digital registration required a working email and phone number. Not all patients had this, or were willing to share their email. Some only had a landline. Online registration page was more suited for completion by clinicians/research assistants, than by patients. The registration interface was small and slow at times, and may cause patients/clinicians to lose interest.	Focus solely on paper consent, and signing at time of encounter, with recording of email if available. A dedicated tray was set up to collect signed paper consent forms for processing. Digital registration completed in back end by PI, with copies of consent forms mailed to patients. Placeholders inserted when email/phone number was not provided.
Clinical workflow integration	Some reported inconvenience to consent patients during on calls and when dealing with emergency patients. Consent forms not readily available when in the emergency department or in theatres. Database considered to be cumbersome, and data entry was difficult and time consuming. Some users reported the double column interface to be confusing and difficult to navigate. Portal link not always readily available due to workstations having location‐based generic login. Website was sluggish at times via hospital internet, and users had to slow down when trying to input data. Some users gave up. Task given a lower priority when workload increases. One user reported lack of mobile computability/mobile application when attempting to access the portal from a mobile device.	Consent forms supplied to ward offices, on call rooms, theatres, clinics. Digital file of the consent form with portal direct link sent to email of all participating medical officers to allow point of care printing of forms and access Advised all participating medical officers to focus on inputting baseline data, with remaining data to be entered retrospectively by PI, based on clinical note entries.

During the second recruitment period at TQEH, 225 patients were identified to have had a clinical encounter regarding their abdominal wall hernia (Table 4). Twenty‐eight patients were excluded due to not speaking English. All patients had the capacity to consent. Of the remaining 197 patients, 74 were encountered by participating clinicians. Twenty‐five patients (34.7%) were enrolled, with two patients declining to participate. The remaining patients did not have a clear, documented reason for non‐enrolment. The true recruitment rate was 12.7%.

**TABLE 4:** Breakdown of enrolled patients at the Queen Elizabeth Hospital, from 07/08/2024 to 02/02/2025 (second period).

Clinical journey TQEH	Subgroups	Available pts	Eligible pts	Seen pts^b^	Pt declined	Pt enrolled	% capture (conditional)	% capture (true)
Outpatients—Non‐operative	All	75	68	19	0	0	0.0%	0.0%
	Watchful waiting	24	21	10	0	0	—	
	Optimisation/prehabilitation	35	32	7	0	0	—	
	General follow up	16	15	2	0	0	—	
Outpatients—Operative	All	123	106	52	2	24	46.1%	22.6%
	Booking only	47	43	27	0	11	—	
	Booking with surgery only^a^	64	54	22	2	11	—	
	Booking with surgery and follow up	12	9	3	0	2	—	
Emergency—Without prior elective booking	All	24	23	3	0	1	33.3%	0.04%
	Surgery only	22	21	3	0	1	—	
	Surgery with follow up	2	2	0	0	0	—	
Combined	Total	225	197	74	2	25	33.8%	12.7%

Abbreviations: Pt, patients; TQEH, the Queen Elizabeth Hospital.

^a^ Some patients had emergency surgery, instead of their intended elective booking.

^b^ Patients seen by participating clinicians.

Over a similar 6‐month period at SVPHS, 119 patients were encountered by participating clinicians (Table 5). Sixty‐one patients declined to participate, and 58 patients were enrolled, achieving a maximum possible conditional recruitment rate of 48.7%. The total number of patients who presented to SVPHS with an abdominal hernia problem could not be determined due to limited access to medical records; as such, the true recruitment rate remained unknown.

**TABLE 5:** Breakdown of enrolled patients at St Vincent's Private Hospital Sydney, from 15/08/2024 to 10/02/2025 and St Vincent's Private Hospital Sydney.

Clinical Journey SVPHS	Subgroups	Available pts	Eligible pts	Seen pts^c^	Pt declined	Pt enrolled	% capture (conditional)	% capture (true)
Outpatients—Non‐operative	All	^a^	^a^	^b^				
	Watchful waiting			—				
	Optimisation/prehabilitation			—				
	General follow up			—				
Outpatients—Operative	All	^a^	^a^	119	61	58	48.7%	^e^
	Booking only			68	61	7	—	
	Booking with surgery only			50	0	50	—	
	Booking with surgery and follow up			1	0	1	—	
Emergency—Without prior elective booking	All	^a^	^a^	0^d^	—	—	—	
	Surgery only			—				
	Surgery with follow up			—				
Combined	Total			119	61	58	48.7%	^e^

Abbreviations: Pt, patients; SVPHS, St Vincent's Private Hospital Sydney.

^a^ True number encountered by other private surgeons was unknown.

^b^ Incomplete data for patients managed non‐operatively.

^c^ Patients seen by participating clinicians.

^d^ All emergencies are admitted and managed by St Vincent's Public Hospital Sydney.

^e^ Cannot be calculated as eligible patients are unknown.

Patients were predominantly recruited by senior clinicians, namely a single surgical fellow (76.0%) for TQEH; a surgical fellow (31.7%) and a consultant (68.3%) at SVPHS (Supplementary S8). Gender, age and socio‐economic status were similar throughout all stages of recruitment within each site (Tables 6 and 7), with insignificant differences (p>0.05) (Supplementary S9). Of the patients recruited between sites during the specified period, SVPHS had a significantly greater male ratio (${Χ}^{2}\left[1\right]=10.31,p<0.001$), older patients (${Χ}^{2}\left[1\right]=12.71,p<0.001$), and better socioeconomic status than TQEH (${Χ}^{2}\left[9\right]=794.81,p<0.001$).

**TABLE 6:** Demographics of enrolled patients at the Queen Elizabeth Hospital, from 07/08/2024 to 02/02/2025 (second period).

TQEH	Available pts	%	Eligible pts	%	Seen pts^a^	%	Enrolled	%
Total	222	100.0	197	100.0	74	100.0	25	100.0
Gender								
Female	85	38.3	74	37.6	21	28.4	7	28.0
Male	137	61.7	123	62.4	53	71.6	18	72.0
Age								
< 65 years	124	55.9	115	58.4	40	54.1	15	60.0
≥ 65 years	98	44.1	82	41.6	34	45.9	10	40.0
Mean (SD)	61.3 (15.6)		60.9 (15.4)		61.2 (16.2)		60.8 (17.2)	
ABS SEIFA relative socio‐economic disadvantage								
Decile 1	51	23.0	43	21.8	14	18.9	4	16.0
Decile 2	7	3.2	7	3.6	2	2.7	1	4.0
Decile 3	41	18.5	36	18.3	10	13.5	0	0.0
Decile 4	9	4.1	9	4.6	4	5.4	2	8.0
Decile 5	38	17.1	37	18.8	13	17.6	8	32.0
Decile 6	30	13.5	24	12.2	8	10.8	2	8.0
Decile 7	17	7.7	14	7.1	4	5.4	0	0.0
Decile 8	12	5.4	12	6.1	8	10.8	2	8.0
Decile 9	14	6.3	12	6.1	9	12.2	4	16.0
Decile 10	3	1.4	3	1.5	2	2.7	2	8.0

*Note:* Decile 1 is top 10% of disadvantage. Decile 10 is bottom 10% of disadvantage (i.e., most privileged).

Abbreviations: ABS SEIFA, Australian Bureau of Statistics – Socio‐Economic Indexes for Areas; Pt, patient; SD, standard deviation; TQEH, the Queen Elizabeth Hospital.

^a^ Patients seen by participating clinicians.

**TABLE 7:** Demographics of enrolled patients at St Vincent's Private Hospital Sydney, from 15/08/2024 to 10/02/2025.

SVPHS	Available pts	%	Eligible pts	%	Seen pts^b^	%	Enrolled	%
Total	^a^		^a^		119	100.0	58	100.0
Gender								
Female	^a^		^a^		18	15.1	6	10.3
Male	^a^		^a^		101	84.9	52	89.7
Age								
< 65 years	^a^		^a^		45	37.8	23	39.7
≥ 65 years	^a^		^a^		74	62.2	35	60.3
Mean (SD)	^a^		^a^		63.5 (18.5)		61.5 (20.4)	
ABS SEIFA relative socio‐economic disadvantage								
Decile 1	^a^		^a^		2	1.7	2	3.4
Decile 2	^a^		^a^		0	0.0	0	0.0
Decile 3	^a^		^a^		2	1.7	2	3.4
Decile 4	^a^		^a^		19	16.0	11	19.0
Decile 5	^a^		^a^		1	0.8	1	1.7
Decile 6	^a^		^a^		8	6.7	5	8.6
Decile 7	^a^		^a^		4	3.4	2	3.4
Decile 8	^a^		^a^		20	16.8	8	13.8
Decile 9	^a^		^a^		12	10.1	3	5.2
Decile 10	^a^		^a^		51	42.9	24	41.4

*Note:* Decile 1 is top 10% of disadvantage. Decile 10 is bottom 10% of disadvantage (i.e., most privileged).

Abbreviations: ABS SEIFA, Australian Bureau of Statistics—Socio‐Economic Indexes for Areas; Pt, patient; SD, standard deviation; SVPHS, St Vincent Private Hospital Sydney.

^a^ True number encountered by other private surgeons was unknown.

^b^ Patients seen by participating clinicians.

Currently, 16 active hernia registries have been described in the global literature (Supplementary S10 and S11) [4, 5, 35, 36, 37, 38, 39, 40, 41, 42, 43, 44, 45, 46, 47, 48, 49, 50, 51, 52, 53, 54, 55, 56, 57, 58, 59, 60, 61]. The majority of registries were located in Europe and the Americas, with some being multinational [4]. Most registries were deidentified, or had identifiable information that could only be accessed by the treating hospital [4, 5, 35, 36, 37, 38, 39, 40, 41, 42, 43, 44, 45, 46, 47, 48, 49, 50, 54, 55, 56, 57, 58, 59]. Data validation processes were commonly present [4, 5, 35, 36, 37, 38, 41, 42, 43, 44, 45, 46, 47, 49, 50, 51, 52, 53, 55, 57, 58, 59, 60]. Only the Danish and Swedish hernia registries have openly reported achieving > 95% patient recruitment, as reporting is required for healthcare reimbursement [4, 35, 36, 41, 42, 43, 44]. Both government‐funded registries have ‘opt‐out’ systems for patient consent, are linked with national patient data and use external validation processes to ensure data accuracy [4, 35, 36, 42, 43, 44].

## 4 Discussion

This study demonstrated that it is possible for metropolitan English‐speaking patients to be enrolled in a hernia CQR by clinicians during clinical encounters. However, in the absence of incentives and sustained funding, clinician goodwill alone was insufficient to achieve the minimum standards required by the Commission for a cost‐effective, representative CQR. Site participation was limited by the need for local clinicians to individually organise site‐specific HREC approvals. Two out of the four intended sites withdrew during the study and limited the number of patients that could be recruited. With only two centres, uncertainty existed as to whether the differences observed between the two sites were due to chance alone or actual observable differences. Whilst efforts were made to attempt to achieve similar recruitment conditions, there are likely site‐specific factors that have not been accounted for.

Ideally, this study should have included non‐English‐speaking CALD patients. Exclusion occurred for practical reasons. The online portal, patient handouts and consent forms had been written and approved by HREC in English. There was no official professional capacity to translate the website, handouts and consent forms into other languages. To participate in the study, all patients were required to provide informed consent due to data privacy concerns expressed by HREC. The inclusion of CALD patients would likely have required the presence of professional medical translators, which was not anticipated to be consistently available. There was no capacity to assign a dedicated research assistant to facilitate the consent process. The exclusion of CALD patients likely introduced a systemic selection bias into the results. However, these biases were unlikely to have significantly altered the poor recruitment rate observed, nor resolved the underlying critical structural problems identified within the CQR.

The study was also limited by the small number of personnel, short duration and non‐blinded non‐registered exploratory study design. Recruitment was likely skewed towards senior clinicians due to the method used to count patients and encounters. The observations made in this study could be due to the individuals involved, and not necessarily be applicable to other clinicians at other healthcare facilities. The variable levels of clinician interest impacted patient recruitment, with highly motivated clinicians recruiting disproportionate levels compared to those with lesser interest. With limited numbers of participating clinicians, not all eligible patients were encountered during normal clinical duties. The conventional, true recruitment rate (total recruited patients divided by total eligible patients) could not be calculated for SVPHS due to incomplete admission data secondary to privacy concerns. A conditional recruitment rate (total recruited patients divided by total eligible patients encountered by participating clinicians) has been provided as a substitute to provide some degree of clarity based on the available information. Even with this unorthodox adjustment, which carried the risk of systemic bias, the conditional recruitment rate during the study period across the two sites remained unacceptably low. No further conclusions could be drawn due to the degree of missing data, but the study revealed critical structural issues in the ANZ Hernia CQR that would necessitate a redesign.

Compared to ANZASM and AOANJRR (Table 8), the aim and scope of the prototype ANZ Hernia CQR are currently very broad without a clear primary endpoint. The CQR attempts to be multi‐role but does not appear to excel in any particular aspect. Built on the foundations of a learning healthcare system (LHS), the ANZ Hernia CQR was intended to be integrated into the clinical workflow so that data may be entered as treatment is provided. The CQR, in the setting of an LHS, is essentially ‘virtual’ because registry data is being summarised from the treatment data that is being entered, such as for complex hernia multidisciplinary meetings. In theory, this could work for small‐scaled operations in a single hospital or local health network. However, local use would limit its ability to draw any conclusions for the rest of Australia and New Zealand. If the CQR was to be binational, then all clinicians across Australia and New Zealand would be required to use this system on a daily basis to input treatment details, in addition to using pre‐existing electronic medical record systems at individual healthcare facilities necessary for medicolegal reasons. One could argue that the use of independent data collectors [4, 65], automated extractions from existing systems or AI‐assisted voice dictation systems could automate certain steps and potentially reduce the burden on clinicians. A clinician could dictate an outpatient or operative note into a secure application, while the background AI extracts from the transcript and prefill the Registry form. These automated processes could substantially reduce the data entry workload traditionally required by clinicians for CQRs and improve overall efficiency and engagement. Yet these solutions require either a variable degree of upfront cost or investment in infrastructure, notwithstanding the technical challenges of collating data from public and private systems across states, territories and nations.

**TABLE 8:** Comparison between ANZASM, AOANJRR and the prototype ANZ Hernia CQR [62, 63, 64].

Name	Australia and New Zealand Audit of Surgical Mortality	Australian Orthopaedic Association National Joint Replacement Registry	Australia and New Zealand Hernia Learning Healthcare System Virtual Clinical Quality Registry
Abbreviated	ANZASM	AOANJRR	ANZ Hernia CQR
State	Active, since 2001	Active, since 1999	Prototype
Aims	To inform, educate, facilitate change, and improve quality of practice within surgery To highlight system and process errors To identify trends in surgical mortality	To define, improve and maintain the quality of care for individuals receiving joint replacement surgery	To be a longitudinal prospective data collection of hernia procedures throughout Australia and New Zealand
Scope	Peer review of all deaths associated with surgical care	Collection of defined minimal data set to enable outcomes to be determined based on patient characteristics, prosthesis type and features, method of prosthesis fixation and surgical technique used	All abdominal wall hernias
Principle outcome measured	Preventable events	Time to first revision surgery	Not defined
Governance	RACS – Research, Audit and Academic Surgery	AOA – Registry Working Group	ANZ Hernia – Database Subcommittee
Funding	Individual State/Territory's Department of Health	Commonwealth Department of Health	ANZ Hernia
Participation	Mandatory	Mandatory	Not defined
Data collection	Structured form (online), with form completion required as part of annual RACS CPD accreditation	Structured form (hard copy), transcribed into system by expert data entry staff. Infrastructure for online form present, but not in use yet.	Structured form (online)
Form length	Size A4, 11 pages, containing 26 major categories of multiple‐choice questions, with room for free text	Size A4, 2 pages, containing 6–8 major categories of multiple‐choice or binary questions, with room for device sticker application and free text. Separate form for each joint type	20 major categories, containing 228 multiple‐choice, binary or free text questions
Data validation	External peer review, via 1st/2nd line assessments, validating against data provided by state/territory health departments	Validated against data provided by state and territory health departments	Not defined
Data management	RACS – data validation, analysis, storage Local state/territory audit offices—data entry, technical support	SAHMRI (contracted)—data entry, validation, analysis, storage Adelaide University/University of South Australia (contracted)—analytical techniques	Data Dissect (contracted)—data storage
Patient consent	Default inclusion. Opt out not stated	Default inclusion. Patient may elect to opt out via informing treating surgeon or contacting Registry	Not defined (currently opt in, due to ethics)
Patient confidentiality	Deidentified, unless to facilitate medical records retrieval for 2nd line assessment	Deidentified	Identifiable on system Deidentified when extracted
Legal protection	Quality assurance activity under the Commonwealth Qualified Privilege Scheme	Quality assurance activity under the Commonwealth Qualified Privilege Scheme	Not defined

Abbreviations: AOA, Australian Orthopaedic Association; CPD, continued professional development; RACS, Royal Australasian College of Surgeons.

More importantly, these solutions do not address the fundamental problems that arise from large case numbers. Since the CQR currently does not exclude non‐operative cases, the estimated annual case numbers are likely greater than the annual operative numbers (> 100 000). To put this into perspective, the ANZASM recorded 9355 deaths over a two‐year period (2021–2022), while AOANJRR reported 151 051 joint replacements (hip, knee and shoulder) in 2025 [14, 62]. Although AOANJRR appears to have similar caseloads to abdominal wall hernias, their data forms are shorter and of much narrower focus, requiring only the completion of a single double‐sided sheet of A4 paper form at the time of operation (simplified further via liberal application of product and patient stickers), as opposed to the currently unprintable ANZ Hernia CQR with 228 questions [62]. It is well known in survey studies that shorter forms achieve better responses and completion by participants [66, 67]. Deploying the ANZ Hernia CQR and its LHS functions in its current configuration across two nations is potentially an expensive, labour‐intensive undertaking that may not necessarily have adequate validated means for independent data validation and quality assurance. It has been consistently shown in the past that excellent data capturing of incomplete patient populations in any clinical registry is a resource‐intensive exercise that is destined to fail from the beginning [4, 10, 50, 68]. Incomplete registry data is additionally at risk of predisposing to systematically misleading conclusions, including under recognition of complications, dilution of safety signals and false reassurances for institutions and funders, and ultimately defeats the purpose of having a CQR in the first place.

If the purpose of the CQR is for device monitoring, then the current CQR is likely overengineered. An LHS structure, beneficial and necessary under specific situations, is not required to report intraoperative events and likely only serves to overburden the average user. Both ANZASM and AOANNJR have demonstrated that a primary enabler of success is to engage clinicians through peer review, rather than data entry, which can be dealt with by delegating the task to independent data management services [14, 62]. Both ANZASM and AOANNJR are mandatory and linked with continued professional development requirements of the respective colleges, thus remain independent of clinician goodwill. Both registries publish national reports on a regular basis and have the options to provide confidential individualised feedback to clinicians for ongoing learning. Independent data management also ensures that the data being entered is of high quality and enables centralised data validation and quality assurance. AOANJRR has demonstrated that their centralised system can facilitate long‐term follow‐up of patient‐reported outcome measures (PROMs), with a respectable completion rate of 74.1% pre‐operatively and 63.0% post‐operatively [62]. Having a clear primary endpoint for CQR may also facilitate better engagement from both clinicians and patients, as opposed to an aimless, short‐lived data collection exercise. Referencing the endpoint used by AOANNJR, a possible primary endpoint for abdominal wall hernias could be time to hernia reoperation. A reoperation would indicate a clinically and functionally significant hernia recurrence has occurred, an undesirable outcome that hernia surgeons would actively avoid. Pertinent information to record in the registry would likely include baseline patient characteristics at the time of operation, details of the hernia (e.g., European Hernia Society classification), details of the operation, adjunct therapy (e.g., botulinum toxin injection) and what mesh device was inserted. Unlike joint replacements, hernias can be repaired with a suture‐only technique without mesh insertion, such as in a contaminated operative field, which may be worth incorporating into the registry despite it not being a mesh device per se. Similar to what AOANNJR has done, forms could be made specific to each type of hernia, such as groin, primary ventral and incisional, minimising the visual distractions and cognitive load on the user during form filling [62]. A hard‐copy form inside the operating theatre transforms the task into a team exercise, such as the application of mesh device tracking stickers by the scout nurse at the time of mesh implantation, as opposed to a clinician‐only task [62].

The shift away from an LHS system also provides a potential solution to the privacy and confidentiality issues raised by HREC. By default, an LHS system retains patient identifiers to ensure future data may be logged as part of clinical encounters. The recording of identifiable information on data servers not located within existing healthcare data infrastructure was a significant point of concern raised by local HREC in the current study. Additional assurances and checks were required to be in place and to ensure confidentiality was not breached, since the collection of identifiable sensitive medical information could be in violation of local privacy laws, such as Section 95A of the Australian Privacy Act 1988 [69, 70]. These privacy risks complicate HREC applications and generate additional hurdles to enrol patients across multiple centres. For mesh device monitoring at a binational level, it is difficult to justify the inclusion and retention of patient identifiers beyond initial data verification purposes to ensure forms are completed correctly. By deidentifying patients, ANZASM and AOANJRR can operate on a default inclusion basis, with options for patients to ‘opt out’ [14, 62]. This likely contributes to the high levels of patient enrolment seen among these two registries and facilitates the inclusion of CALD patients, which are consistent with previous observations of ‘opt‐out’ systems [71]. From a clinician's point of view, certain clinicians may feel uncomfortable having their details retained in an LHS due to the potential legal repercussions. Both the ANZASM and AOANJRR have been assessed as quality assurance activities and are legally protected by the Commonwealth Qualified Privilege Scheme, Part VC of the Health Insurance Act 1973, so that any data recorded within registries are for educational purposes only and may never be used for legal proceedings against any individual. This has been reported by the Australian Government to encourage engagement from patients and health professionals, resulting in better quality data that is necessary to improve health services [72]. Provided that there is no breach of privacy laws or confidentiality clauses, there may be an argument to log entries under the patient's Australian Medicare Number or New Zealand's National Health Index (NHI), in some ways similar to the Danish National Patient Register [38, 42]. Both numbers are lifelong non‐identifiable alphanumeric strings unique to the individuals of the respective countries, which are readily recorded within both public and private sectors. This could provide a reliable, confidential avenue for reverse searching to identify what mesh implants had been inserted into a specific patient 10–20 years ago.

The main cost driver of registries is independent data management, which has also been the primary enabler of the success seen in ANZASM and AOANJRR [14, 62]. Stable long‐term funding and dedicated data infrastructure that is independent of clinician goodwill ensure registries will continue to function over time, a feature that is particularly critical to monitoring abdominal wall hernia outcomes. Hernia registries often fail after early enthusiasm wanes and soft funding ceases, with previous studies reporting perceptions of increased administrative burden voiced by Dutch surgeons in a national survey [47], user compliance problems reported by the New England Veteran Affairs Hernia Registry and the South African Hernia Interest Group of South Africa registry [40, 56], and the long‐term attrition and waning interest described by the Brazilian HERNIACLINIC‐QoL registry [68]. ANZASM is funded by the Department of Health of the respective states and territories, and AOANJRR is funded directly by the federal Department of Health, Disability and Aging [14, 62]. In comparison, the prototype CQR only has limited funding from the ANZ Hernia Society, with a future vision of securing federal funding, provided the CQR can demonstrate capabilities to improve patient healthcare in Australia and New Zealand. For absolute objectivity, the CQR should not be directly funded by the biomedical device industry, as this may introduce sponsorship bias and invalidate the registry's data (Supplementary S9) [73]. Health economic analysis of AOANJRR has previously demonstrated being highly cost‐effective, with a benefit–cost ratio of 10.29 [74]. Similar analyses will need to be performed for the Hernia CQR once it has been redesigned.

Hernia treatment in Australia and New Zealand continues to remain heterogenous, partly due to the lack of high‐quality evidence‐based guidelines [75]. Hernia implants are regulated at sales and distribution by the Australian Therapeutic Goods Administration (TGA) and New Zealand Medicines and Medical Devices Safety Authority (MEDSAFE), but there is currently no uniform means to report or investigate patient outcomes post implantation, unless an adverse event notification is made to the regulatory authorities. The lack of transparency or early warning system for potentially malfunctioning mesh devices places patients at an elevated risk that is no longer acceptable in the current digital age of evidence‐based medicine. A device‐focused ANZ Hernia CQR could be the answer to this problem, bringing objective, clinically relevant transparency to the safety and outcomes of hernia mesh devices across the two nations. As reported by other CQRs [4], a high representative recruitment rate of patients is likely not possible without mandatory participation, as demonstrated by the Danish and Swedish registries. Logging entries into the redesigned CQR could be mandated as compulsory reporting by clinicians when claiming treatment reimbursement via the Australian Medical Benefits Schedule (MBS) or the New Zealand Pharmaceutical Management Agency/Te Pātaka Whaioranga (PHARMAC) [76, 77]. For example, the Australian MBS item numbers for bariatric surgery (e.g., 31 569, 31 572, 31 575, 31 581) currently require entries to be made into the Monash Bariatric Surgery Registry before surgeons can receive government reimbursement for providing healthcare services (i.e., bariatric surgery). A similar government mandate may need to be introduced for hernia meshes. Although the CQR could provide valuable system level benchmarking, it is not designed for real time clinical governance or has the capacity for root cause analysis. Participation in a hernia CQR does not remove the obligation of clinicians, local departments or health networks to conduct regular scheduled audits, morbidity and mortality meetings and peer‐reviewed reflective practices that can and should provide more immediate, targeted, relevant, achievable and measurable feedback to the clinician or treating team.

Certain international hernia registries have used participation as an incentive for accreditation and certification purposes [4, 58]. Whilst these practices may have a role in their country of origin, it is largely not appropriate for Australia and New Zealand if objectivity is to be preserved. Clinician engagement is important, but clinician comfort or interest should not determine the limits of data integrity where patient safety and accountability are concerned. In the absence of universal participation (100%) and representative data capture, registries cannot function as surrogate accreditation mechanisms, confer professional status, justify differential practice models or tolerate unmonitored practice by nonparticipants while increasing scrutiny for those who voluntarily participate.

This study has identified inherent structural challenges within the prototype ANZ Herna CQR prior to the investment in significant resources and has provided a rare second chance to reset expectations and redesign the CQR. The proposed ANZ Hernia CQR redesign is based on the experiences obtained by the two most successful surgical CQRs in Australia. The critiques presented here are not to invalidate the collective efforts and contributions of project members, participating clinicians and patients, but to present a critical reflection of the difficulties of implementing a CQR for a common, heterogeneous surgical condition. The need to monitor hernia mesh devices and their clinical outcomes cannot be understated. If meaningful, system‐wide quality improvement is to be delivered as envisioned, the CQR will require deliberate adjustment.

The current system may not be optimal, but the digital infrastructure that has been set up so far remains a readily available and configurable platform that could be updated to address the identified issues. Paper‐based reporting could function as a low‐cost test bed in the interim that is available for immediate trial in clinical settings, provided an independent registry support team can be established via securement of a stable funding stream. A clear minimum dataset will need to be clearly defined that captures critical information, does not burden the collector, and facilitates data quality audits. Once the paper‐based system matures, the entry forms and experiences obtained may better inform the design and structure of a digital interface, along with the benefit of a tiered introduction of the redesigned ANZ Hernia CQR to clinicians.

The future of CQRs will likely shift from a paper‐based format towards digital, particularly with the recent introduction and widespread inclusion of AI‐power functions. Some of these changes are already occurring, with ANZASM migrating their paper‐based surgical case forms to an online platform where fellows can access and complete at any time. Likewise, AOANJRR has setup digital infrastructure to allow online reporting, though not formally in use yet. AI‐assisted functions are being integrated into various Australian and international cancer databases [78, 79], with preliminary results suggestive of a 60%–80% reduction in time spent on performing data cleaning activities [80]. The University of New South Wales' Centre for Big Data Research in Health recently secured a highly competitive medical research future fund (MRFF) to develop and deploy a software framework for next generation clinical registries via the use of common data models, AI and cloud‐based computing [81]. There will likely be some time before AI can be sufficiently intelligent and reliable, that is, no data hallucinations, overfitting or misinterpretation, that the system may safely automate and replace human inputs within CQRs [82]. Any planned use of advanced software for the ANZ Hernia CQR will need to take into consideration the limited resources and infrastructure that are available in regional, rural and remote areas, which currently accounts for 27% of Australians and 19% of New Zealanders [83, 84]. Stakeholder meetings may need to be organised with binational representation of clinicians, nursing and patient representatives from various levels of the healthcare system, including patients from CALD backgrounds, Aboriginal and Torres Strait Islander peoples, and Māori people. The governance of the future ANZ Hernia CQR will need to be clear and align with the national quality and safety frameworks of both Australia and New Zealand. There is much work that lies ahead before the ANZ Hernia CQR can become an active registry across Australia and New Zealand.

## Author Contributions

**Edward Young:** conceptualization, methodology, software, investigation, validation, formal analysis, data curation, writing – original draft, writing – review and editing, resources, funding acquisition, visualization, project administration. **Doug Fenton‐Lee:** conceptualization, methodology, software, investigation, validation, formal analysis, resources, data curation, writing – review and editing, project administration, supervision, funding acquisition. **Julian Süsstrunk:** methodology, investigation, validation, resources, data curation, writing – review and editing. **Jorgen Ferguson:** methodology, investigation, validation, resources, data curation, writing – review and editing. **Matthew Amprayil:** methodology, investigation, validation, resources, data curation, writing – review and editing. **Yijie Yin:** methodology, investigation, validation, resources, data curation, writing – review and editing. **Rabieh Abu Assi:** investigation, methodology, validation, writing – review and editing, data curation, resources. **Alex Karatassas:** conceptualization, methodology, writing – review and editing, resources, supervision, funding acquisition. **Sanjeev Khurana:** conceptualization, methodology, software, resources, writing – review and editing, supervision. **Chrys Hensman:** conceptualization, methodology, validation, writing – review and editing, funding acquisition, supervision. **Andrew Tse:** methodology, investigation, resources, data curation, writing – review and editing. **Hamish Urquhart:** investigation, methodology, writing – review and editing, data curation, resources. **Reginald V. N. Lord:** investigation, methodology, writing – review and editing, data curation, resources. **Jean Wong:** conceptualization, methodology, software, writing – review and editing, supervision. **Darren Tonkin:** conceptualization, methodology, software, writing – review and editing, supervision. **Markus Trochsler:** conceptualization, methodology, software, writing – review and editing, supervision. **Guy J. Maddern:** validation, resources, writing – review and editing, funding acquisition, supervision.

## Funding

This work was supported by Australian Government Research Training Program; The Hospital Research Foundation (THRF) Higher Degree by Research Top‐up Scholarship; The Hospital Research Foundation (THRF) Group Project Grant, 2024‐S‐RETRO‐002‐QA 25249; ANZ Hernia Society.

## Ethics Statement

The study was approved by the Central Adelaide Local Health Network (CALHN) Human Research Ethics Committee (HREC) for the Queen Elizabeth Hospital (Reference No. 15562), with reciprocal ethics approved by St Vincent's Hospital HREC for extension to St Vincent's Private Hospital Sydney. The study was performed in accordance with the ethical standards laid down in the Declaration of Helsinki.

## Conflicts of Interest

E.Y. is a recipient of the Australian Government Research Training Program (RTP) Scholarship and the Hospital Research Foundation (THRF) Higher Degree by Research Top‐up Scholarship. G.J.M. is a recipient of the Hospital Research Foundation (THRF) Group Project Grant (2024‐S‐RETRO‐002‐QA 25249). The ANZ Hernia Registry is funded in whole by the ANZ Hernia Society and is hosted by Data Dissect Pty Ltd. A.K., C.H., D.F. are board members of the ANZ Hernia Society CQR and Database Subcommittee. S.K. is a co‐founder of Data Dissect Pty Ltd. D.F. is a recipient of a Medtronic Education Grant at St Vincent Public and Private Hospital Sydney, which provided funds to employ J.F. G.J.M. is a Speciality Editor for the Quality section in the ANZ Journal of Surgery.

## Supporting information


**Supplementary S1:** New patient registration.
**Supplementary S2:** Clinical data—Baseline.
**Supplementary S3:** Clinical data—Planning.
**Supplementary S4:** Clinical data—Treatment.
**Supplementary S5:** Clinical data—Follow up.
**Supplementary S6:** Infographic handout.
**Supplementary S7:** Search strategy.
**Supplementary S8:** Breakdown of enrolments by clinician role during each recruitment period at the Queen Elizabeth Hospital.
**Supplementary S9:** Statistical comparison of demographics between subgroups and recruitment site.
**Supplementary S10:** Hernia registries identified from the literature.
**Supplementary S11:** Characteristics of identified hernia registries.

## Data Availability

The data that supports the findings of this study are available in the main text and Supporting Information of this article.
