# Supplementary material for: Tale of Two Centres—Exploring Bedside Challenges to Implementing a Clinical Quality Registry for Abdominal Wall Hernias in Metropolitan Australia
Source: ANZ J Surg. 2026 Apr 24;96(7-8):2142–57. doi: 10.1111/ans.70694 (PMC13536264; doi:10.1111/ans.70694)
Supplement: Supplementary file 1 [file ANS-96-2142-s001.docx]

# Supplementary Legend

Supplementary 1 – New Patient Registration

Supplementary 2 – Clinical Data – Baseline

Supplementary 3 – Clinical Data – Planning

Supplementary 4 – Clinical Data - Treatment

Supplementary 5 – Clinical Data – Follow up

Supplementary 6 – Infographic handout

Supplementary 7 – Search Strategy

Supplementary 8 – Breakdown of enrolments by clinician role during each recruitment period at the Queen Elizabeth Hospital

Supplementary 9 – Statistical comparison of demographics between subgroups and recruitment site

Supplementary 10 – Hernia registries identified from the literature

Supplementary 11 – Characteristics of identified hernia registries

# Supplementary 1

New Patient Registration

For ANZ Hernia Clinical Quality Registry. All items mandatory, unless otherwise specified. For readability, some questions have been shortened from its original state.

|  | Item | Options |
| --- | --- | --- |
|  | Consent |  |
| 1 | Consent to collection and storage of identifiable information | Yes / No |
| 2 | Consent to inclusion of de-identified information for research | Yes / No |
| 3 | Signature | Interface for signature |
|  | Patient details |  |
| 4 | Full name | Free text |
| 5 | Date of birth | Free text |
| 6 | Medical record number | Free text |
| 7 | Postcode | Free text |
| 8 | Gender | Male / female / free-text |
| 9 | Email | Free text |
| 10 | Mobile number | Free text |
|  | Health assessment |  |
| 11 | Estimated weight, kg | Free text |
| 12 | Estimated height, cm | Free text |
| 13 | History of smoking | Yes / No |
| *14 | Frequency of smoking | Ex / rarely / monthly / weekly |
| *15 | Use of other forms of nicotine products | Never / e-cigarette / vape / patch / gum |
| *16 | Use of non-nicotine e-cigarette / vape | Yes/No |
| *17 | Frequency of non-nicotine e-cigarette / vape use | Ex / rarely / monthly / weekly |
| 17 | Frequency of alcohol use | Never / once monthly / 2-4 times monthly / 2-4 times weekly / >4 weekly / daily |
| 18 | Diabetes | Yes/No |
| *19 | Type of diabetes | 1 / 2 |
| 20 | Renal failure, requiring dialysis | Yes / No |
| 21 | COPD / emphysema / chronic bronchitis / asthma | Yes / No |
| 22 | Cancer, past or active | Yes / No |
| 23 | Previous abdominal / pelvic operations | Yes / No |
| 24 | Chronic pain | Yes / No |
| 25 | Medications | No / anticoagulants / corticosteroids / chemotherapy / immunosuppressants / inhalers / analgesia |
| 26 | Clinical frailty scale, self-assessment | Very fit / well / managing well / vulnerable / mildly frail / moderately frail / severely frail / very severely frail / terminally ill |
| 27 | Exercise frequency | Never / monthly / weekly / daily / unsure |
| 28 | Functional status | Independent / partially independent / dependent |

* Optional questions that may be left blank

COPD: chronic obstructive pulmonary disease

# Supplementary 2

Clinical Data – Baseline

For ANZ Hernia Clinical Quality Registry. All items mandatory, unless otherwise specified. For readability, some questions have been shortened from its original state.

|  | Item | Options |
| --- | --- | --- |
|  | Patient details |  |
| 1 | Full name | Prepopulated |
| 2 | Date of birth | Prepopulated |
| 3 | Medical record number | Prepopulated |
| 4 | Postcode | Prepopulated |
| 5 | Gender | Prepopulated |
| 6 | Email | Prepopulated |
| 7 | Mobile number | Prepopulated |
| *8 | Mailing address | Free text |
| *9 | Country of birth | Free text |
| *10 | Aboriginal or Torres Strait Islander Status | One / both / none |
| *11 | Primary language | Free text |
|  | General Health |  |
| 12 | Weight, kg | Free text |
| 13 | Height, cm | Free text |
| 14 | BMI, kg/cm^2^ | Auto populated |
| 15 | History of smoking | Yes / No |
| 16 | Frequency of smoking | Ex / rarely / monthly / weekly |
| 17 | Use of other forms of nicotine products | Never / e-cigarette / vape / patch / gum |
| 18 | History of alcohol use | Yes / No |
| *19 | Frequency of alcohol use | Never / once monthly / 2-4 times monthly / 2-4 times weekly / >4 weekly / daily |
| *20 | Standard drinks per day | 1-2 / 3-4 / 5-6 / 7-9 / 10+ |
| 21 | Cancer | Yes / No |
| *22 | Active cancer | Yes / No |
| 23 | Mental health diagnosis | Yes / No |
| *24 | Mental health conditions | Major depression / anxiety disorder / free text |
| 25 | Psychotropic drug use | Yes / No |
| 26 | Opioid use | Yes / No |
| *27 | Opioid use history | Within 90 days of prescribed opioids / Over 90 days of prescribed opioids / Over 90 days of non-prescribed opioids |
| 28 | Functional Status | Independent / partially independent / dependent |
| 29 | Exercise frequency | Never / monthly / weekly / daily / unsure |
|  | Comorbidities |  |
| 30 | Diabetes | Yes / No |
| *31 | HbA1c value | Free text |
| 32 | Hypertension | Yes / No |
| 33 | Dialysis | Yes / No |
| 34 | Creatinine, μmol/L | Free text |
| *35 | Dialysis type | Haemodialysis / peritoneal |
| 36 | Liver failure | Yes / No |
| *37 | Bilirubin, μmol/L | Free text |
| *38 | INR | Free text |
| 39 | Ascites | Yes / No |
| *40 | Ascites type | Hepatic / malignant / unknown |
| 41 | Inflammatory bowel disease | Ulcerative colitis / Crohn’s disease |
| 42 | Dyspnoea | Yes / No |
| 43 | Anti-platelet medication | Yes / No |
| 44 | Anticoagulant medication | Yes / No |
| 45 | Immunosuppressants | Yes / No |
| *46 | Type of immunosuppressant | Corticosteroid / immunomodulators / chemotherapy |
| 47 | Abdominal aortic aneurysm | Yes / No |
| 48 | Collagen vascular disease | Yes / No |
| *49 | Type of collagen vascular disease | Systemic lupus erythematosus / scleroderma / Ehlers-Danlos syndrome / Marfan’s syndrome / Other |
| 50 | Congestive heart failure | Yes / No |
|  | Previous hernia surgery |  |
| 51 | Hernia type | Ventral / groin / diastasis recti |
| *52 | IF ventral, which type | Primary / incisional / parastomal |
| *53 | IF groin, which type | Inguinal / femoral / obturator / other |
| *54 | History of surgery | < 3 months / 3-12 months / > 12 months |
| 55 | History of component separation | Yes / No |
| 56 | History of open abdomen hernia repair | Yes / No |
| 57 | History of abdominal wall surgical site infection | Yes / No |
| *58 | Infection location | Superficial / deep / organ space |
| *59 | Active infection | Yes / No |
| 60 | History of solid organ transplantation | Yes / No |
| *61 | If transplant, which type | Donor / recipient |
| 62 | History of surgery for cancer | Yes / No |
| *63 | If cancer, which type | Colorectal / urological / gynaecological / other / unknown |
| 64 | Previous pelvic operation | Yes / No |
| *65 | Other pelvic operations | Free text |
|  | Baseline outcome assessments |  |
| 66 | Patient reported experience measures (PREM) | Designated questionnaire |
| 67 | Patient reported outcome measures (PROM) | Designated questionnaire |

* Optional questions that may be left blank

INR: international normalised ratio

# Supplementary 3

Clinical Data – Planning

For ANZ Hernia Clinical Quality Registry. All items mandatory, unless otherwise specified. For readability, some questions have been shortened from its original state.

|  | Item | Options |
| --- | --- | --- |
|  | Patient Prehab Survey |  |
| 1 | Mental health issues impacting on preparation / recovery from surgery | Yes / No |
| 2 | GP aware of mental health issue and helping to manage | Yes / No |
| 3 | Suffer from pain that has lasted 12+ weeks | Yes / No |
| 4 | GP aware of chronic pain and helping to manage | Yes / No |
| 5 | Chronic pain under control | Yes / No |
| 6 | Drink alcohol / take strong pain killers / use recreational drugs | Yes / No |
| 7 | Use strong pain kills >1 per week | Yes / No |
| 8 | Used recreational drugs in past 1 year | Yes / No |
| 9 | History of anaemia / iron deficiency | Yes / No |
| 10 | Physical activity status | Fit and active / moderately active / little active / not active |
| 11 | Can walk 300 metres without aids and without resting | Yes / No |
| 12 | Can walk up 10 steps without aids and without resting | Yes / No |
| 13 | Use of mobility aids | Yes / No |
| *14 | Mobility aid, type | Stick / frame / wheelchair / other |
| *15 | Duration of mobility aid, in months | Free text |
| 16 | Feeling of fatigue | None / little / some / most / all the time |
| 17 | Five or more illnesses | Yes / No |
| 18 | Unplanned weight loss >5kg or >5% of body weight | Yes / No |
| 19 | Mechanical falls, in past 1 year | Yes / No |
| 20 | Bone fracture, in past 3 years | Yes / No |
| 21 | Diabetes | Yes / No |
| *22 | Diabetes management | None / diet / medication |
|  | Pre-surgery plan |  |
| 23 | Follow-up required | Yes / No |
| 24 | Follow-up date | Free text |
| 25 | Surgery booking urgency | Cat 1 – less than 4 weeks / Cat 2 – within 3 months / Cat 3 – within 1 year / Cat 4 – deferred |
| 26 | Current surgery booking status | On wait list / scheduled |
| 27 | Admission date | Free text |
| 28 | Ventral hernia working group (VHWG) grade | Grade 1 – low risk / Grade 2 – comorbid / Grade 3 – potentially contaminated / Grade 4 – infected |
| 29 | Carolina’s Equation for Determining Associated Risks (CeDAR) score | Free text |
| 30 | Patient preoperative optimisation | Nutritional counselling / exercise / BMI < 30 / smoking cessation / glycaemic control HbA1c < 7.2% / Frailty assessment / preperitoneal progressive pneumoperitoneum (PPP) / botulinum injection |
| 31 | Botulinum toxin (Botox) injection | Yes / No |
| *32 | Injection date | Free text |
| *33 | Botulinum toxin brand | Free text |
| *34 | Muscles injection | Transversus abdominis / internal oblique / external oblique |
| *35 | Side of injection | Left / right / bilateral |
| 36 | Action plan | Free text |

* Optional questions that may be left blank

BMI: body mass index

# Supplementary 4

Clinical Data - Treatment

For ANZ Hernia Clinical Quality Registry. All items mandatory, unless otherwise specified. For readability, some questions have been shortened from its original state.

|  | Item | Options |
| --- | --- | --- |
|  | Peri-op |  |
| 1 | Nature of surgery | Emergency / Elective |
| 2 | Date of surgery | Free text |
| 3 | Surgeon in charge / bed card | Free text |
| 4 | Operating surgeon | Free text |
| 5 | Assistant | Free text |
| 6 | Preoperative chlorhexidine scrub at home | Yes / No |
| 7 | Bowel preparation | Yes / No |
| *8 | Bowel preparation type | Mechanical / Oral antibiotic |
| 9 | ASA score | Free text |
| 10 | Hernia reducible under anaesthetics | Completely / Partially / None |
| 11 | Prophylactic antibiotics given within 1 hour prior to incision | Yes / No |
| 12 | Intraoperative wound status | Clean / Clean-contamination / Contaminated / Dirty |
|  | Anaesthesia |  |
| 13 | Type | General / Sedation / Spinal / Local |
| *14 | If Local, type | Lidocaine / Bupivacaine / Liposomal bupivacaine |
| 15 | Epidural for analgesia | Yes / No |
| 16 | Transversus abdominis plane (TAP) block | Yes / No |
| 17 | Rectus sheath block | Yes / No |
|  | Operative details - General |  |
| 18 | Hernia type | Ventral / groin / diastasis recti |
| 19 | European Hernia Society (EHS) classification | Free text |
| 20 | Procedure notes | Free text |
| 21 | Intraoperative complications | Yes / No |
| *22 | If complications, type | Haemorrhage / peritoneal access injury / bowel injury / bladder injury / liver injury / gastric injury / major vascular injury / spermatic cord injury / testicular injury |
| 23 | Concomitant procedures | Yes / No |
| *24 | If concomitant procedures, type | Hernia / foregut / endocrine / hepatobiliary / pancreatic / small intestine / colorectal / obstetric / gynaecology /urology / soft tissue / plastics |
|  | Operative details - Ventral |  |
| 25 | Type | Primary / incisional / parastomal |
| 26 | Indication | Pain / enlarging hernia / bowel obstruction / fistula / infected mesh / incarcerated bowel |
| 27 | Approach | Open / laparoscopic / laparoscopic assisted open / robotic / robotic assisted open / minimal invasive converted to open / endoscopic |
| 28 | Repair technique | Onlay / MILOS / Rives-Stoppa / TAR / TARM with TAR / eTEP / Preperitoneal / IPOM / IPOM+ / LIRA / TAPP / rTARM with rTAR / reTEP / rTAPP / rIPROM / SCOLA / TESAR(RR) / ELAR / eMILOS |
| 29 | Access | Open cut down / Veress-blind trocar / Veress-optical trocar / optimal trocar |
| 30 | Subcutaneous flaps raised | Yes / No |
| 31 | Hernia length, cm | Free text |
| 32 | Hernia width, cm | Free text |
| 33 | Hernia width code | W1 – <4 cm \| W2 – 4-10 cm \| W3 – <10 cm |
| 34 | Stoma present | Yes / No |
| *35 | If stoma present, disposition | Left in situ / re-matured at same location / moved to new site / ostomy taken down |
| 36 | Contents of sac | Live / spleen / kidney / small bowel / large bowel / lipoma / extraperitoneal fat / other |
| 37 | Hernia sac – lateral | Left in situ / reduced intact / excised and reduced |
| 38 | Hernia sac – medial | Left in situ / reduced intact / excised and reduced |
| 39 | Myofascial release | Yes / No |
| *40 | If myofascial release, which type | External oblique / transversus abdominis / anterior rectus sheath incision / posterior rectus sheath incision/ |
| *41 | If myofascial release, surgical method | Open / endoscopic |
| *42 | If myofascial release, laterality | Unilateral / bilateral |
| 43 | Chemical component separation | Yes / No |
| 44 | Closure of fascia | Yes / No |
| *45 | If closure, type of suture | Absorbable / permanent / mesh |
| *46 | If closure, technique | Running / interrupted / mattress |
| 47 | Wound closure | Staple / suture / glue / strip / wet to dry packing / wick / negative pressure wound therapy |
| *48 | If negative pressure wound therapy, specify brand | Free text |
| 49 | Drain used | Yes / No |
| 50 | Number of drains | Free text |
|  | Operative details - Groin |  |
| 25 | Which type | Inguinal / femoral / obturator / other |
| 26 | Laterality | Left / right / bilateral |
| 27 | Level of complexity | Simple / Complex |
| 28 | Indication for repair | Asymptomatic / painful bulge / inguinal canal disruption / core muscle injury / chronic groin pain / bowel obstruction / infected mesh / incidental contralateral hernia found on operation |
| 29 | Operative approach | Open / laparoscopic / robotic / lap converted to open / Robotic converted to open / other |
| 30 | Operative method | Lichtenstein / Shoudice / Desarda / TAPP / TIPP / TREPP / TEP / eTEP / rTAPP / rTEP / reTEP / |
| 31 | Operative findings – Lateral | 0 – No hernia  1 – < 1.5 cm  2 – 1.5-3.0 cm  3 – > 3.0 cm |
| 32 | Operative findings - Medial | 0 – No hernia  1 – < 1.5 cm  2 – 1.5-3.0 cm  3 – > 3.0 cm |
| 33 | Cord lipoma | Yes / No |
| 34 | Other findings | Small bowel / large bowel / omentum / bladder / pre-peritoneal fat / other |
| 35 | Hernia sac – lateral | Left in situ / reduced intact / excised and reduced |
| 36 | Hernia sac – medial | Left in situ / reduced intact / excised and reduced |
| 37 | Posterior wall repair | Yes / No |
| *38 | If posterior wall repair, suture type | Absorbable / permanent |
| *39 | If posterior wall repair, closure technique | Running / interrupted / figure of eight / mattress / self-locking |
| 40 | Individual nerves identified | Yes / No |
| *41 | Ilioinguinal nerve | No action / excised |
| *42 | Iliohypogastric nerve | No action / excised |
| *43 | Genital branch of genitofemoral nerve | No action / excised |
| *44 | Femoral branch of genitofemoral nerve | No action / excised |
| *45 | Lateral femoral cutaneous nerve | No action / excised |
| *46 | Round ligament transection (females only) | Yes / No |
|  | Operative details – Diastasis recti |  |
| 25 | Diastasis length, cm | Free text |
| 26 | Diastasis width, cm | Free text |
| 27 | Diastasis width code | W1, <3cm \| W2, ≥3-5cm \| W3, ≥5cm |
|  | Mesh Details |  |
| 51 | Date of implantation | Free text |
| 52 | Scan barcode | Barcode scanner |
| 53 | Device ID | Free text |
| 54 | Brand name | Free text |
| 55 | Version / Model | Free text |
| 56 | Device description | Free text |
| 57 | Device serial number | Free text |
| 58 | Device expiry date | Free text |
| 59 | Device batch / lot number | Free text |
| 60 | Device size | Free text |
| 61 | Company name | Free text |
| 62 | Mesh application | Plug / Flat / Plug and Patch / Anatomic |
| 63 | Mesh fixation | Sutures / Tacks / Glue / Staples / None (self-fixing) |
|  | Post-op and discharge |  |
| 64 | Disposition | Discharge / Return to ward |
| 65 | Prescription opioid analgesia | Oxycodone / hydromorphone / codeine / methadone / morphine / meperidine / tapentadol / tramadol / fentanyl |
| 66 | Dosage, mg | Free text |
| 68 | Number of tablets prescribed | Free text |
| 69 | Non-opioid analgesia | Paracetamol / ibuprofen / naproxen / meloxicam / celecoxib / pregabalin / gabapentin / aspirin |
| 70 | Physical therapy referral | Yes / No |
| 71 | Abdominal binder | Yes / No |
| 72 | Time off work | Yes / No |
| 73 | Discharge advice | Free text |

* Optional questions that may be left blank

# Supplementary 5

Clinical Data – Follow up

For ANZ Hernia Clinical Quality Registry. All items mandatory, unless otherwise specified. For readability, some questions have been shortened from its original state.

|  | Item | Options |
| --- | --- | --- |
|  | Post-op follow up |  |
| 1 | Date | Free text |
| 2 | Type | Pre-discharge / 7 day / 30 day / 3 month / 6 month / yearly / additional |
| 3 | Method | In person / phone / telehealth |
| 4 | Date of discharge from repair | Free text |
| 5 | Length in ICU | Free text |
| 6 | Total length of stay | Free text |
| 7 | Readmission | Yes / No |
| 8 | Reason for readmission | Pain / prosthetic related complication / wound complication / bleeding complication / thrombotic non-cardiac complication / cardiac complication / respiratory complication / gastrointestinal complication / other |
| 9 | Surgical site infection | Yes / No |
| *10 | If surgical site infection, type | Superficial / deep / organ |
| *11 | If surgical site infection, treatment | No treatment / PO antibiotics / IV antibiotics / wound opening / wound debridement / suture excision / percutaneous drainage / partial mesh removal / complete mesh removal / negative pressure therapy |
| 12 | Surgical site occurrence | Yes / No |
| *13 | If surgical site occurrence, type | Wound cellulitis / non-healing incisional wound / fascial disruption / skin or soft tissue ischaemia / skin or soft tissue necrosis / wound serous drainage / wound purulent drainage / chronic sinus drainage / localised stab wound infection / stitch abscess / seroma / infected seroma / haematoma / infected haematoma / exposed biologic mesh / exposed synthetic mesh / contaminated synthetic mesh /infected biologic mesh / infected synthetic mesh / mucocutaneous anastomosis disruption / enterocutaneous fistula / unspecified surgical site occurrence |
| *14 | If surgical site occurrence, treatment | No treatment / PO antibiotics / IV antibiotics / wound opening / wound debridement / suture excision / percutaneous drainage / partial mesh removal / complete mesh removal |
| 15 | Other complications | Ileus / bowel obstruction / pain requiring intervention / pulmonary embolism / stroke / sepsis / septic shock / myocardial infarction / cardiac arrest / urinary retention / urinary tract infection / progressive renal insufficiency / acute renal failure / pneumonia / postoperative respiratory failure / ventilator / coma / peripheral nerve injury / post op bleeding transfusion / graft prothesis flap failure / death |
| 16 | Clavien-Dindo classification | Free text |
| 17 | Pian management |  |
| 18 | Prescription opioid analgesia | Oxycodone / hydromorphone / codeine / methadone / morphine / meperidine / tapentadol / tramadol / fentanyl |
| 19 | Dosage, mg | Free text |
| 20 | Number of tablets prescribed | Free text |
| 21 | Non-opioid analgesia | Paracetamol / ibuprofen / naproxen / meloxicam / celecoxib / pregabalin / gabapentin / aspirin |
|  | Follow up outcome assessments |  |
| 22 | Inclusion in automated reminder | Yes / No |
| 23 | Start date for follow up | Free text |
| 24 | Patient reported experience measures (PREM) | Designated questionnaire |
| 25 | Patient reported outcome measures (PROM) | Designated questionnaire |

* Optional questions that may be left blank

# Supplementary 6

Infographic handout

# Supplementary 7

Search strategy

| Database | Search Syntax |
| --- | --- |
| PubMed | (((implement*[Title/Abstract]) OR (recruit*[Title/Abstract])) AND (((registr*[Title/Abstract]) OR (database*[Title/Abstract])))) AND (hernia[Title/Abstract])  ((database*[Title/Abstract]) OR (registr*[Title/Abstract])) AND (hernia[Title/Abstract]) |
| Scopus | (((TITLE-ABS-KEY (implementation) OR TITLE-ABS-KEY (recruit)) AND (TITLE-ABS-KEY (registry) OR TITLE-ABS-KEY (database))) AND TITLE-ABS-KEY (hernia))  ((TITLE-ABS-KEY (database) OR TITLE-ABS-KEY (registry)) AND TITLE-ABS-KEY (hernia)) |

# Supplementary 8

Breakdown of enrolments by clinician role during each recruitment period at the Queen Elizabeth Hospital and St Vincent’s Private Hospital Sydney

| Site | Period | Role | Pt discussed | Pt declined | Pt enrolled |
| --- | --- | --- | --- | --- | --- |
| TQEH | 22/07/2024 – 02/08/2024 | Consultant 1 | 0 | - | - |
|  |  | Consultant 2 | 0 | - | - |
|  |  | Fellow 1 | 2 | 1 | 0 |
|  |  | Fellow 2 | 0 | 1 | 0 |
|  |  | Registrar 1 | 1 | 0 | 1 |
|  |  | RMO 1 | 0 | 0 | 1 |
|  | 07/08/2024 – 02/02/2025 | Consultant 1 | 0 | - | - |
|  |  | Consultant 2 | 0 | - | - |
|  |  | Fellow 1 | 19 | 0 | 19 |
|  |  | Fellow 2 | 2 | 0 | 2 |
|  |  | Registrar 2 | 2 | 0 | 2 |
|  |  | Registrar 3 | 1 | 0 | 1 |
|  |  | Registrar 4 | 0 | - | - |
|  |  | Registrar 5 | 0 | - | - |
|  |  | RMO 2 | 1 | 0 | 1 |
|  |  | RMO 3 | 0 | - | - |
|  |  | Intern 1 | 0 | - | - |
|  |  | Intern 2 | 0 | - | - |
|  | 02/02/2025 – 16/02/2025 | Consultant 1 | 0 | - | - |
|  |  | Consultant 2 | 0 | - | - |
|  |  | Fellow 3 | 0 | - | - |
|  |  | Registrar 6 | 0 | - | - |
| SVPHS | 15/08/2024 – 10/02/2025 | Consultant A | 41 | 0 | 41 |
|  |  | Consultant B | 0 | - | - |
|  |  | Consultant C | 0 | - | - |
|  |  | Consultant D | 0 | - | - |
|  |  | Consultant E | 0 | - | - |
|  |  | Fellow A | 79 | 60 | 19 |

TQEH: the Queen Elizabeth Hospital

SVPHS: St Vincent’s Private Hospital Sydney

RMO: resident medical officer

# Supplementary 9

Statistical comparison of demographics between subgroups and recruitment site

| Group | Comparison | Statistical Test | p-value |
| --- | --- | --- | --- |
| TQEH |  |  |  |
| Gender, portions | Seen pts, vs. available | $X^{2}(1)=3.08$ | $0.079$ |
|  | Enrolled pts, vs. seen | $X^{2}(1)=0.01$ | $0.967$ |
| Age, portions | Seen pts, vs. available | $X^{2}(1)=0.10$ | $0.749$ |
|  | Enrolled pts, vs. seen | $X^{2}(1)=0.35$ | $0.551$ |
| SEIFA | Seen pts, vs. available | $X^{2}\left( 9 \right)=11.81$ | $0.224$ |
|  | Enrolled pts, vs. seen | $X^{2}(9)=13.84$ | $0.128$ |
| SVPHS |  |  |  |
| Gender, portions | Seen pts, vs. available | - | - |
|  | Enrolled pts, vs. seen | $X^{2}(1)=1.03$ | $0.310$ |
| Age, portions | Seen pts, vs. available | - | - |
|  | Enrolled pts, vs. seen | $X^{2}(1)=0.08$ | $0.773$ |
| SEIFA | Seen pts, vs. available | - | - |
|  | Enrolled pts, vs. seen | $X^{2}(9)=5.07$ | $0.751$ |
| Between sites |  |  |  |
| Gender, portions | Seen pts, SVPHS vs. TQEH | $X^{2}(1)=10.31$ | $<0.001$ |
| Age, portions | Seen pts, SVPHS vs. TQEH | $X^{2}(1)=12.71$ | $<0.001$ |
| SEIFA | Seen pts, SVPHS vs. TQEH | $X^{2}(9)=794.81$ | $<0.001$ |

TQEH: the Queen Elizabeth Hospital

SVPHS: St Vincent Private Hospital Sydney

Pt: patient

SEIFA: Socio-Economic Indexes for Areas

# Supplementary 10

Hernia registries identified from the literature

| Country | Name of Registry | Organisation | Active | Funding | Participating Centres | Hospitals | Estimated Catchment Population | Inclusion | Opt in / out consent | Ethics | Reference |
| --- | --- | --- | --- | --- | --- | --- | --- | --- | --- | --- | --- |
| Denmark | Danish Hernia Database (DHDB) | Steering Committee of the Danish Hernia Database | 1998 onward | Danish Government | Mandatory, all hospitals, all eligible procedures | 32 | 6.0 million | Age >18 years, elective + emergency operations, inguinal | Opt out | Not required by Danish law | ^4, 41, 42, 44, 55^ |
| Sweden | Swedish Hernia Registry (SHR) | Registry steering group | 1992 onward | Swedish National Board of Health and Welfare | Voluntary | 90 | 10.6 million | Adults, elective+ emergency operations, inguinal | Opt out | Gothenburg University Regional Ethical Board | ^4, 35, 36^ |
| Germany; Austria; Switzerland | Herniamed Registry | Herniamed, a network of surgeons | 2009 onward | Industry Funding: PFM medical, Storz, FEG, Bard, Ethicon, Braun, MenkeMed, Dahlhausen, Medtronic | Voluntary | 640 | 69.6 million | Inguinal, primary ventral, incisional, parastomal, hiatal | Opt in | Unclear | ^4, 84^ |
| France | Club Hernie (CH) | Group of surgeons | 2011 onward | Industry Funding: Medtronic, FEG, Bard, Ethicon | Voluntary | 70 | Unclear | Inguinal, primary ventral, incisional, parastomal | Opt in | Unclear | ^4, 58^ |
| Europe | EuraHS | European Hernia Society (EHS) | 2012 onward | Industry Funding: Medtronic, FEG, Bard, Ethicon | Voluntary | Unclear | Unclear | Ventral incisional, parastomal, hiatal, inguinal, open abdomen, abdominal closures, prophylactic | Opt in | Local ethics board for each user | ^4, 48^ |
| Spain | Registro Espanol de Eventraciones (EVEREG) | Abdominal Wall and Sutures Division of the Spanish Association of Surgeons | 2012 onward | Industry Funding: B. Braun | Voluntary | 113 | 32.3 million | All ages, post-laparotomy hernias, recurrence post-incisional or ventral hernia repair | Opt in | Ethics Committee of each participating hospitals | ^4, 49, 50^ |
| United States / Brazil / Canada | Americas Hernia Society Collaboration Registry (AHSQC) | Americas Hernia Society (AHS) | 2013 onward | Industry Funding: Bard, Allergan, Intuitive, Medtronic, W. L. Gore | Voluntary | 594 | 110.9 million | Inguinal, primary ventral, parastomal | Opt in | Unclear | ^4^ |
| Brazil | HERNIACLINIC-QoL | Oswaldo Cruz German Hospital | 2024 | Voluntary | Voluntary | 1 | Unclear | Inguinal, ventral | Opt in | Oswaldo Cruz German Hospital Ethics Committee | ^45^ |
| South Africa | Hernia Interest Group of South Africa (HIG[SA]) | South African Society of Endoscopic Surgeons (SASES) | 2019 | Not-for-profit HIG(SA) | Voluntary | Unclear | Unclear | Age >18, inguinal, ventral, parastomal, prophylactic mesh | Opt in | University of Cape Town | ^39, 40^ |
| China | Chinese Hernia Registry | Chinese Hernia Surgery Alliance | 2017 | Chinese Hernia Surgery Alliance | Voluntary | 518 | Unclear | All ages, ventral, inguinal, parastomal, hiatal | Opt in | Unclear | ^52, 53^ |
| Japan | National Clinical Database (extended) | Japan Hernia Society | 2021 | National Clinical Database and Industry Funding: Intuitive Surgical Sarl, Johnson and Johnson, Nipro Co. | Voluntary | Unclear | Unclear | Groin hernias | Opt in | Unclear | ^51^ |
| United States | Michigan Surgical Quality Collaborative Core Optimization Hernia Registry (MSQC-COHR) | Blue Cross Blue Shield | 2020 | Private Payer: Blue Cross Blue Shield of Michigan | Voluntary | 70 | 10.1 million | All ages, ventral and inguinal hernias | Opt in | University of Michigan Institutional Review Board | ^46, 57^ |
| United States | New England Veteran Affairs Hernia Registry (NEVAHR) | Veteran Affairs New England Workgroup | 2011 | Veteran Affairs New England Healthcare System | Voluntary | Unclear | 15.1 million | All ages, ventral hernias | Opt in | Veteran Affairs Boston Institutional Review Board; Research and Development Committee | ^56^ |
| Italy | Italian Hernia Club Registry | Centre for Research on Health and Social Care Management (CERGAS) | 2015 | Confindustria Dispositivi Medici Servizi Srl | Voluntary | Unclear | Unclear | All ages, hernia repair with Bard Phasix or Bard Phasix ST | Opt in | Bocconi University | ^54^ |
| United Kingdom | British Hernia Society Registry | British Hernia Society | 2025 | Industry funding | Voluntary | Pilot period | Unclear | All ages, all abdominal wall hernia surgeries | Opt in | National Health Service Health Research Authority | ^37^ |
| Russia | Hernia of Abdominal Wall Russian Quality Registry (AWRORA) | Russian Society of Herniologists / Russian Society of Surgeons | 2018 | Private funding by authors + external donors | Voluntary | 120+ | Unclear | All ages, all hernia surgeries, including hiatal | Opt in | Sokolov North-Western District Scientific and Clinical Centre | ^60^ |

# Supplementary 11

Characteristics of identified hernia registries

| Country | Name of Registry | Data Entry ?Who | Data entry ?how | Accept missing data? | Privacy | Systems integration | % of all hernias in country / region | Post-entry audit? | Incentive | Reference |
| --- | --- | --- | --- | --- | --- | --- | --- | --- | --- | --- |
| Denmark | Danish Hernia Database (DHDB) | Surgeon; register secretary / nurse | Online (previous paper) | No | National patient code | Danish National Patient Register (DNPR) | 95% | Data cross-referenced with DNPR, with register secretary / nurse containing surgeons if discrepancy | No | ^4, 41, 44, 55^ |
| Sweden | Swedish Hernia Registry (SHR) | Surgeon | Online | No | Deidentified | Swedish Personal Identity Numbers; Swedish Population Registry | 98% | Random external validation | No | ^4, 35, 36^ |
| Germany; Austria; Switzerland | Herniamed Registry | Surgeon | Online | No | No, but can only be seen by treating institution | Standalone | 15-20% | 1st year, self-validation and check. After 3 years, random external validation | Prerequisite for certification as hernia centre from German Hernia Society | ^4, 84^ |
| France | Club Hernie (CH) | Surgeon; research assist | Online | No | Deidentified | Standalone | 2-3% | Clinical research assistant performs review and validation | Continuing medical education credits | ^4, 58^ |
| Europe | EuraHS | Surgeons; | Online | Yes | Deidentified | Standalone | Unknown | Personal data validation | Certificate for registration from EuraHS | ^4, 48^ |
| Spain | Registro Espanol de Eventraciones (EVEREG) | Surgeons; | Online | No | Deidentified | Standalone | Unknown | Annual monitoring by an Executive Committee | No | ^4, 49, 50^ |
| United States / Brazil / Canada | Americas Hernia Society Collaboration Registry (AHSQC) | Surgeon; | Online | No | Deidentified | Standalone | Unknown | Systematic data assurance, including completion and accuracy | Certificate from American Board of Surgery | ^4^ |
| Brazil | HERNIACLINIC-QoL | Surgeon | Online | Yes | Deidentified | Standalone | Unknown | Research personnel | No | ^45^ |
| South Africa | Hernia Interest Group of South Africa (HIG[SA]) | Surgeon | Online | Yes | No, but can only be seen by treating clinician | Clinical notes platform | Unknown | No | No | ^39, 40^ |
| China | Chinese Hernia Registry | Surgeon | Online | Yes | Unclear | Standalone | Unknown | No | No | ^52, 53^ |
| Japan | National Clinical Database (extended) | Surgeon | Online | Unclear | Unclear | Standalone | 37% | No | Board certification | ^51^ |
| United States | Michigan Surgical Quality Collaborative Core Optimization Hernia Registry (MSQC-COHR) | Surgeon | Online | No | Deidentified | Standalone | Unknown | Regular inter-rater reliability assessment to check validity and reliability of data | No | ^46, 57^ |
| United States | New England Veteran Affairs Hernia Registry (NEVAHR) | Surgeon; designed nurse reviewer | Online | Yes | Deidentified | Standalone | 37.5% | No | No | ^56^ |
| Italy | Italian Hernia Club Registry | Surgeon | Online | Unclear | Deidentified | Standalone | Unknown | Unknown | Unknown | ^54^ |
| United Kingdom | British Hernia Society Registry | Surgeon | Online | Unclear | Deidentified | Standalone | Unknown | Unknown | Unknown | ^37^ |
| Russia | Hernia of Abdominal Wall Russian Quality Registry (AWRORA) | Surgeon | Online | Yes | No, but can only be seen by treating clinician | Standalone | Unknown | Every two years by the Board, with automated error reports generated at shorter intervals | Unknown | ^60^ |
